# Effects of different compost amendments on the abundance and composition of *alkB* harboring bacterial communities in a soil under industrial use contaminated with hydrocarbons

**DOI:** 10.3389/fmicb.2014.00096

**Published:** 2014-03-13

**Authors:** Stefanie Wallisch, Tjasa Gril, Xia Dong, Gerd Welzl, Christian Bruns, Ester Heath, Marion Engel, Marjetka Suhadolc, Michael Schloter

**Affiliations:** ^1^Research Unit Environmental Genomics, Helmholtz Zentrum MünchenMunich, Germany; ^2^Organic Agricultural Sciences, University of KasselWitzenhausen, Germany; ^3^Jožef Stefan InstituteLjubljana, Slovenia; ^4^Biotechnical Faculty, Center for Soil and Environmental Science, University of LjubljanaLjubljana, Slovenia

**Keywords:** alkane monooxygenase *alkB*, compost, contaminated soils, bioremediation, next generation sequencing

## Abstract

Alkane degrading microorganisms play an important role for the bioremediation of petrogenic contaminated environments. In this study, we investigated the effects of compost addition on the abundance and diversity of bacteria harboring the alkane monooxygenase gene (*alkB*) in an oil-contaminated soil originated from an industrial zone in Celje, Slovenia (Technosol). Soil without any amendments (control soil) and soil amended with two composts differing in their maturation stage and nutrient availability, were incubated under controlled conditions in a microcosm experiment and sampled after 0, 6, 12, and 36 weeks of incubation. As expected the addition of compost stimulated the degradation of alkanes in the investigated soil shortly after the addition. By using quantitative real-time PCR higher number of *alkB* genes were detected in soil samples amended with compost compared to the control soils. To get an insight into the composition of *alkB* harboring microbial communities, we performed next generation sequencing of amplicons of *alkB* gene fragment. Richness and diversity of *alkB* gene harboring prokaryotes was higher in soil mixed with compost compared to control soils with stronger effects of the less maturated, nutrient poor compost. The phylogenetic analysis of communities suggested that the addition of compost stimulated the abundance of *alkB* harboring Actinobacteria during the experiment independent from the maturation stage of the compost. A*lkB* harboring γ-proteobacteria like *Shewanella* or *Hydrocarboniphaga* as well as α-proteobacteria of the genus *Agrobacterium* responded also positively to the addition of compost to soil. The amendment of the less maturated, nutrient poor compost resulted in addition in a large increase of *alkB* harboring bacteria of the Cytophaga group (*Microscilla*) mainly at the early sampling time points. Our data indicates that compost amendments significantly change abundance and diversity pattern of *alkB* harboring microbes in Technosol and might be a useful agent to stimulate bioremediation of hydrocarbons in contaminated soils.

## Introduction

Since almost 150 years industry has been using petroleum-based products as major source of energy (OPEC, 2012). Therefore, the number of industrialized as well as remote areas that show a serious degree of contamination with substances which derive from petroleum is continuously increasing, as the risk for accidental spills or leaks during oil exploration, the industrial manufacturing processes and transport is high (SOER, 2010). Linear and branched alkanes, cycloalkanes and other aromatic compounds being part of petroleum are then released to the environment and have contaminated soil ecosystems as well as water bodies (Pinedo et al., 2013).

However, alkanes are also naturally produced by many living organisms. Alkane derivatives are for example part of plant waxes (Eglinton et al., 1962), pheromones produced by animals (Mori, 2007) or fungal spores (Oró et al., 1966; Fisher et al., 1972). Thus, many microbes are able to degrade alkanes either as mean of detoxification or as source of energy (Ayala and Torres, 2004). Depending on the length of the carbon chain, different types of alkane hydroxylases have been described so far: Alkanes with one to four carbon residues (C_1_–C_4_) are mainly degraded by the methane monooxygenases (Jiang et al., 2010) as well as propane- (Steffan et al., 1997) and butane oxygenases (Dubbels et al., 2007); alkanes with medium chain length (C_6_–C_11_) are metabolized by the alkane monooxygenase system (Kloos et al., 2006), which is mainly found amongst bacteria or by the fungal cytochrome P450 enzyme system (Van Beilen and Funhoff, 2005); long chain alkanes (>C_20_) are preferably transformed by LadA and other hydroxylases (Ji et al., 2013). One organism can harbor several alkane degrading enzyme systems that are activated depending on the quality of the alkanes (Rojo, 2009).

Microbes play a very important role in the development of remediation strategies of sites contaminated with alkanes. However, mainly in soils with technical origin (Technosols) the potential to degrade alkanes after a contamination with petroleum is low. This might be a result of the overall low microbial biomass and activity as a consequence of missing nutrients (Scalenghe and Ferraris, 2009). Furthermore, the low levels of natural alkanes due to the spare vegetation cover might have prohibited the development of an alkane degrading microflora in Technosols (Séré et al., 2008). Compost material, which is mainly based on plant derived litter material, contains high numbers of microbes that are capable to degrade hydrocarbons (Keeling et al., 1994). Therefore, in the past it has been investigated in several studies, if the addition of compost material may stimulate the degradation of pollutants like hydrocarbons in Technosols (e.g., Beaudin et al., 1999; Van Gestel et al., 2003). However, despite the often reported positive effects of compost amendments on the degradation of hydrocarbons in Technosols, it is not clear if these positive effects of compost addition are related to the introduced microbes and their genetic potential to degrade alkanes or to a general shift of microbial community structure in soil as a result of extra nutrients provided by the compost.

Therefore, we investigated in this study the role of composts on the abundance and diversity of alkane degrading microbes in an alkane contaminated Technosol in a laboratory study. We used two contrasting types of composts of different maturation stages, which are characterized by contrasting nutrient levels, to investigate the influence of the compost type on the obtained data. Soil samples were taken at selected time points after the amendment of compost to soil. As a model for alkane degraders, microbes where chosen, which carry the *alkB* gene. This gene codes for a subunit of the bacterial alkane monoxygenase. *AlkB* harboring microbes have been described on the one hand as major players in the degradation of plant derived alkanes (Giebler et al., 2013) and have been detected also in high numbers in soils contaminated with petroleum or crude oil on the other hand (Andria et al., 2009). Thus, this group of microbes might be of interest, if composts are used to stimulate bioremediation of alkanes in contaminated soils. For the analysis of *alkB* diversity an amplicon based pyrosequencing pipeline was performed using extracted DNA from the different samples as well as established primer systems. Abundance of *alkB* harboring bacteria was assessed from the same extracts using the same primers by quantitative real time PCR.

## Materials and methods

### Compost and soil material

The composts used in this experiment were differing from each other and composed from yard waste compost and a mix of yard waste and biowaste from separated organic household waste. While the yard waste type (C1) was mainly based on shredded shrubs and trees the second compost (C2) was consisting of organic kitchen waste (food residues) grass clippings, shredded shrubs and other woody material, vegetable and flower residues. C1 was processed for 1 year at a commercial composting plant in the north of Germany (AHA Hannover-Lahe). Temperature development during the composting period was up to 65°C. The windrow was turned regularly according to the schedule of the composting plant each week in the first 8 weeks with decreasing frequency in dependence of temperature, water, and oxygen content [not less than 15% (vol.)]. The compost was stable and typical for this type of yard waste compost in terms of nutrient contents [C_org_ 14%, N_org_ 1.01%, available (mineral) N 230, P 715, and K 4006 mg kg^−1^, pH 7.4]. Compost C2, was sampled already after 2 weeks of composting of a model compost windrow (2 m^3^) reaching temperature peaks of 72°C in this phase of high decomposition. Carbon loss of the raw material in the very early period was fairly high: the starting material had a C/N-ratio of 41 (33.4% C_org_, 0.8% N_org_) but after already 14 days of composting the carbon content decreased to 29.3% and N_org_ increased relatively to 0.94% (C/N-ratio 31). The heap was turned once after 1 week processing. The available (mineral) nutrients of C2 were fairly low as expected (N 20.8, P 597 and K 3259 mg kg^−1^, pH 7.6)

The soil, showing the typical features of a Technosol, was sampled up to a depth of 20 cm from an industry zone in Celje, Slovenia [46.2335°(N), 15.2764°(E)] in June 2009. The soil has been characterized using ISO referenced standard methods as loamy sand with a C_org_ of 8.7% and a N_tot_ of 1.2%. The pH of the soil was 7.3. The site has been exposed for 150 years of zinc smelting and accompanied chemical industry and it is known for high contamination of soils with hydrocarbons.

### Soil microcosm incubation

Cylinders made of stainless steel with a diameter of 10 cm and heights of 13 cm were used. Thirty-six of these microcosms were hand-packed with 120 g of fresh, homogenized and 5 mm sieved soil (which is equivalent to approximately 100 g dw^−1^ soil). The field bulk density was adjusted to 1.3 g cm^−3^. After preincubation of all microcosms for 1 week at 14°C (reflecting the annual middle temperature of this region) at constant water content [80% of water holding capacity (WHC)], three different treatments were set up: (1) original soil (Soil), which served as a control, (2) soil, mixed with 2.3 g dw^−1^ stable compost (soil + C1), and (3) soil mixed with 2.3 g dw^−1^ young compost (soil + C2). Composts were added to soil in pots after soil conditioning, and toughly mixed with soil. The control soil was also mixed, however, without any addition of amendments. All pots were covered with perforated cups, and kept at 14°C and dark for the whole duration of the experiment. At regular time intervals, aeration and soil water adjustment (80% of WHC) was performed. Sampling was performed at four time points: (i) on the day of experiment set up (week 0), and at (ii) 6 (week 6), (iii) 12 (week 12), and (iv) 36 (week 36) weeks after compost amendment by removing all soil from the microcosm and mixing. Samples of 2 g were transferred to dry ice immediately after sampling and stored at −80°C. For the determination of alkane concentrations, additionally soil samples were taken and air dried. Experiments were performed in three replicates for each treatment and sampling time point.

### Extraction and determination of n-alkanes

Alkanes were extracted from soil samples by Soxhlet extractor (Barnstead/LAB-LINE multi-unit extracting heater, Labline Thermo Scientific, Dubuque, USA) (EPA 3540C, 1996; http://www.epa.gov/osw/hazard/testmethods/sw846/pdfs/3540c.pdf). Briefly 1g of air-dried sample was mixed with 200 μL of internal standard [n-tetracosane-d50 (98%, 75 μg/mL, Cambridge Isotope Laboratories, Andover, MA, USA)], placed into extraction thimble (30 × 100 mm, Macherey-Nagel GmbH&Co. Kg, Düren, Germany) and covered with pre-extracted glass wool (silane untreated, Supelco, Bellefonte, PA, USA). After adding 120 mL of dichloromethane (J.T. Baker, Deventer Nederland), samples were Soxhlet extracted at 60°C for 16 h. After reducing extract volume under gentle stream of nitrogen, solvent was exchanged to cyclohexane (4 mL, Merck, Darmstadt, Germany) and finally reduced to 1 mL in volume. A clean-up on silica gel (grade 12, 28–200 mesh, Sigma Aldrich, Saint Louis, USA) followed. The extract containing alkanes was eluted with 25 mL pentane and reduced in volume to 10 mL. One microliter of sample was injected into gas chromatograph coupled to mass spectrometry detector (HP 6890 Series, Hewlet-Packard, Waldbron, Germany) equipped with Agilent DB-5MS, 30m × 0.25 mm × 0.25 um capillary column. Temperature program was as follows: initial temperature 40°C, rate of 8°C/min to do 300°C (held for 15 min) and carrier gas velocity (He 6.0) was 36 cm/s. Injector temperature and interface temperature were 250 and 280°C, respectively. 17 n-alkanes TRPH (500 μg/mL each, Restek, Florida, USA) were used as external standard.

### DNA extraction

Genomic DNA was extracted from all samples in triplicates (0.5 g soil each) using the Fast DNA Spin Kit for Soil (MP Biomedicals, USA) according to the supplier's manual. Extracted DNA was quantified spectro-photometrically with a NanoDrop 1000 device (NanoDrop Technologies, USA) and visualized with agarose gel electrophoresis (1% agarose stained with ethidium bromide).

### Quantitative real-time PCR of the *alkB* gene

A SYBR Green based quantitative real-time PCR were carried out on a 7300 Real-Time PCR System (Applied Biosystems, Germany) using the primer pair alkB-1f 5′-AAYACIGCICAYGARCTIGGICAYAA-3′ and alkB-1r 5′-GCRTGRTGRTCIGARTGICGYTG-3′ (Kloos et al., 2006; Schulz et al., 2012; Giebler et al., 2013). The expected amplicon size was in the range of 550 bp. In a preliminary test, dilution series of the DNA extracts were performed to avoid inhibition of PCR, resulting in an optimal dilution of 1:50 for all DNA samples (data not shown). Serial plasmid dilutions (carrying the *alkB* gene from *Pseudomonas putida* Gpo1) from 10^6^ to 10^1^ gene copies μl^−1^ served as standard (for details see Schulz et al., 2012). qPCRs were performed in five technical replicates for each sample. The PCR master mix with a total volume of 25 μl contained 0.5 μl 50 mM MgCl_2_, 0.5 μl 10 μM *alkB* specific primers, 0.5 μl 3% bovine serum albumin, 12.5 μl Power SYBR Green Master Mix (Applied Biosystems, Germany) and 2 ng DNA. After a initial denaturation step (10 min 95°C) a touchdown PCR was performed (5 cycles of 45 s 95°C, 1 min 62°C (stepwise reduced to 57°C) and 45 s 72°C, followed by 40 cycles of 45 s 95°C, 1 min 57°C and 45 s 72°C; final extension was 10 min at 72°C). Specificity of the amplified products was tested with a melting-curve and a 1% agarose gel stained with ethidium bromide. The amplification efficiency of 85% was calculated according to the equation

(1) $$\text{Eff = }\left[10^{\left(-1/\text{slope}\right)}-1\right]*100.$$

### Amplicon sequencing of *alkB* fragements

For amplicon sequencing the same primer system as described above was used. *alkB* gene amplicons with unique identifiers each for the forward and reverse strand were sequenced bidirectionally on a 454 GS FLX Titanium platform (Roche, Penzberg, Germany) according the manual for amplicon sequencing provided by Roche. In brief, *alkB* amplicons were generated in three technical replicates using FastStart High Fidelity PCR System (Roche, Germany) in a 50 μl reaction volume containing 1X PCR buffer with 1.8 mM MgCl_2_, 0.2 mM dNTPs, 2.5 U High Fidelity polymerase, 0.2 μM of each primer and 100 ng of template DNA. The PCR reactions were carried out in a thermal cycler (Biometra GmbH, Germany) with the following cycling conditions: initial denaturation (95°C, 10 min) followed by 30 cycles of denaturation (95°C, 45 s), annealing (57°C, 60 s), and elongation (72°C, 45 s), ended with a final extension (72°C, 10 min). The primers were linked to unique identifiers (MIDs) for multiplexing purpose, to a four base library key and to an adaptor site according the instructions of Roche. All PCR products were purified with AMPure Beads (Beckman Coulter, Germany) and subsequently quantified with the Quant-iT PicoGreen dsDNA Assay Kit (Invitrogen, Germany). Amplicon length of 550 bp was determined with a Bioanalyzer 2100 device using a DNA 7500 chip (Agilent Technologies, Germany). All generated amplicons for each sample were pooled in equal concentration. The following emulsion PCR and sequencing of the pool was performed according to Roche's recommendations for amplicons.

An initial signal processing for amplicons was performed with the software GSRunProcessor v.2.6 (Roche, Penzberg, Germany). Subsequently data were analyzed using the software MOTHUR v.1.27.0 (www.mothur.org Schloss et al., 2009) and the online tool FunGene (http://fungene.cme.msu.edu//FunGenePipeline/ as of 2013-07-01, Cole et al., 2009).

For analyzing the community of bacteria harboring *alkB* gene fragments, all forward sequencing data was first trimmed and denoised using the software MOTHUR following the data analysis as described by Schloss et al. (2011). For data analysis, each sample in the pool was identified by its unique MID. Only sequencing reads longer than 200 bp were further processed starting with the dereplicator tool provided by FunGene to remove duplicate sequences. Table S1 (supplementary data) summarizes the number of reads before and after trimming as well as the average read lengths. Chimeric sequences were identified by using UCHIME (Edgar et al., 2011) implemented in the FunGene pipeline and ignored for further analysis. DNA sequences were translated into amino acid sequences and frame shift errors were corrected. After aligning the amino acid sequences by using the HMMER3 algorithm based on the profile hidden markov models (Finn et al., 2011) the sequences were clustered to Operational Taxonomic Units (OTUs, 97% sequence identity) and representative amino acid sequences for each cluster were calculated. Rarefaction curves were created and evenness was calculated according to Shannon (1948). With the software R v.2.13.1 a heat map of the most abundant OTUs containing at least 50 sequences was calculated to determine which OTUs are part of the bacterial communities of each sample. Representative amino acid sequences of the OTUs presented in the heat map were identified via a NCBI BLASTP search against the non-redundant protein sequences GenBank database. All sequences gained from amplicon sequencing were submitted to the sequence read archive (SRA) of GenBank and can be found under the accession number SRP029181.

The phylogenetic analysis based on the *alkB* gene reference database provided by FunGene which contains at present 2012 sequences for this gene. Based on these reference sequences, a phylogenetic tree on amino acid level was constructed using the maximum parsimony algorithm implemented in the software package ARB (Ludwig et al., 2004). Only those 1380 reference sequences that are flanked by the *alkB* primers alkB-1f and alkB-1r were considered. Representative sequences of each OTU except those consisting of singletons were aligned to the reference sequences and added to the tree using the parsimony algorithm implemented in ARB. To make the tree more concise, sequences closely related to reference sequences were grouped to clusters. If necessary, amino acid sequences of specific clusters were identified performing a BLASTP search as described above.

### Statistical analysis

Statistical analysis of quantitative real-time PCR was done performing a variance analysis with treatments and sampling time points as independent factors. Variance homogeneity was tested with the coefficient of Levene. Testing significant differences between the treatments and sampling time points was carried out using a *Post-hoc* test following the Tukey method. All significant differences were calculated based on ANOVA analysis with *p*-values < 0.05 and performed with SPSS v11 (SPSS Inc., USA).

Statistical analysis of the 454 data was performed with the software R v2.13.1 (http://www.R-project.org/) based on a dissimilarity matrix calculated according to the Yue and Clayton theta index of OTUs (containing at least 10 sequences) assigned to each sample. Multivariate statistics was calculated using the packages “vegan” with data transformed by Hellinger (Ramette, 2007) and similarity of bacterial communities was then determined by principal component analysis based on the dissimilarity matrix. Using the tool “adonis” provided by the vegan package, a permutational multivariate analysis of variance (PERMANOVA) was performed based on Euclidean distance calculations.

## Results

### Alkane dynamics

The amount of alkanes in the original soil was in the range of 800–850 mg/kg (Table S2). During the incubation period the alkane concentration decreased in all treatments (also in the control treatments without compost addition); however, the compost addition resulted in higher degradation rates compared to the controls. Whereas after 12 weeks of incubation in the control microcosms concentration of alkanes was reduced to about 640 mg/kg, in the soil samples amended with compost concentrations of alkanes were measured in the range of 500–540 mg/kg. A differing influence of the two compost types was not visible.

### Abundance of *alkB* genes in soil

**Figure 1:**
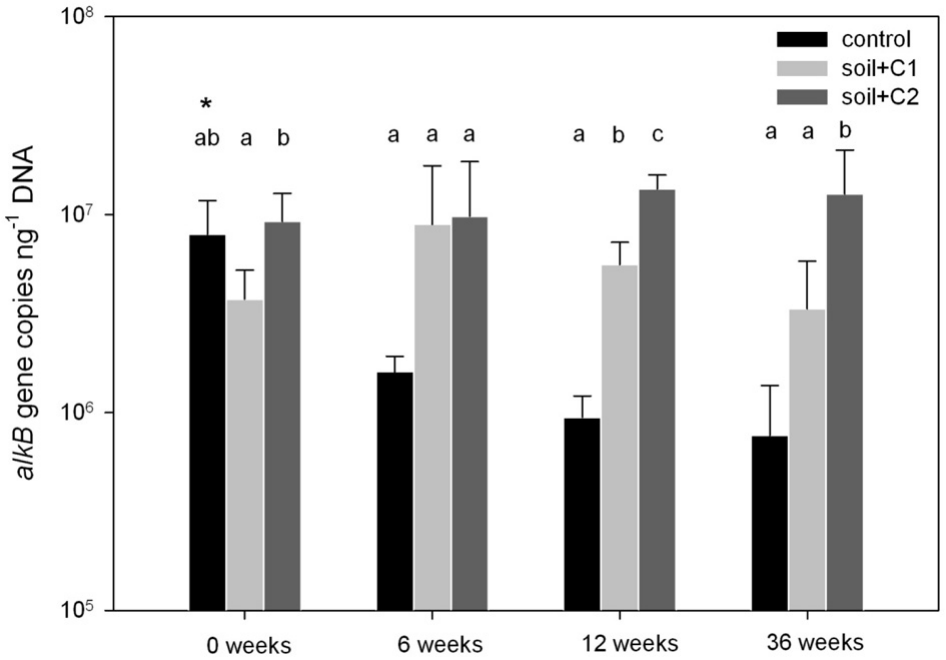
**Gene copy numbers of *alkB* related to ng DNA for different soil treatments**. Significant different treatments within one sampling time point are indicated with different letters, the asterisk indicates significant differences over time within one treatment (*n* = 5, *p*-value < 0.05 based on ANOVA analysis). Sampling after 6 weeks revealed no significant differences due to high standard deviations (indicated by error bars).

As a result of the increase in microbial biomass after compost addition to the soil samples, also *alkB* gene copy numbers were higher in the treatments amended with compost compared to the control treatments (data not shown). To investigate if the compost addition stimulated *alkB* harboring microbes more than others, data were related to the amount of extracted DNA, which was used as a proxy for microbial biomass. The number of *alkB* gene copies in all treatments ranged from 7.65 × 10^5^ to 1.35 × 10^7^ copies ng DNA^−1^ (Figure 1). For the treatments with control soil without amendment of compost, gene copy numbers decreased over time from 7.92 × 10^6^ copies ng DNA^−1^ at the beginning of the experiment (0 weeks) to 7.65 × 10^5^ copies/ng DNA after 36 weeks. In contrast in both treatments with compost addition the number of *alkB* gene copies was constant over the experimental period of 36 weeks or even slightly increased and ranged from 5.59 × 10^6^ copies/ng DNA (soil + C1) to 9.79 × 10^6^ copies/ng DNA (soil + C2). Except for the sampling time point after 6 weeks of incubation, where gene copy numbers between both treatments amended with compost C1 or C2 did not differ, at all other time points *alkB* gene copy numbers in treatments with compost C2 were significantly higher compared to samples where compost C1 has been added.

### Diversity of *alkB* genes in soil

**Figure 2:**
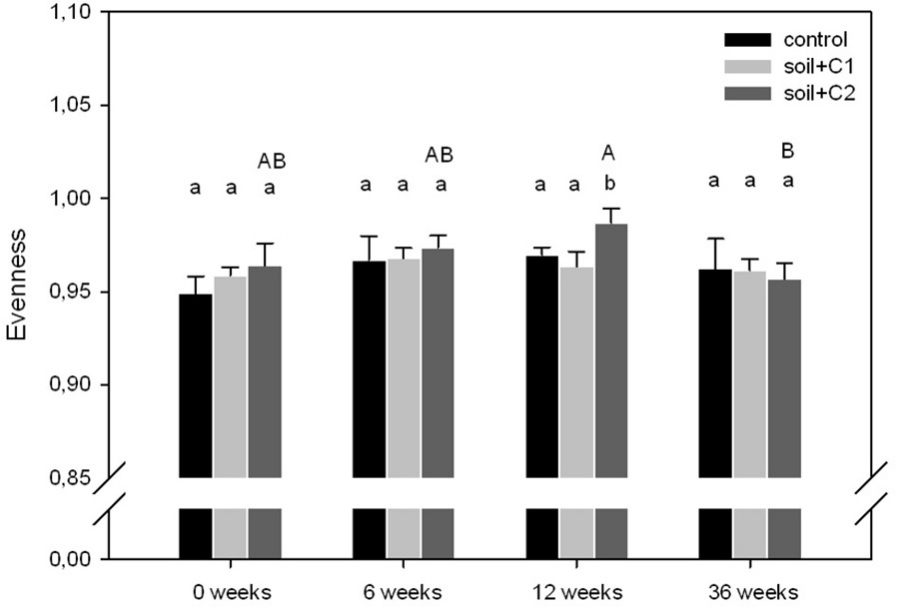
**Evenness of *alkB* gene harboring soil bacteria**. Significant different treatments within one sampling time point are indicated with different lower case letters, the upper case letters indicate significant differences over time within one treatment (*n* = 3, *p*-value < 0.05 based on ANOVA analysis, error bars indicate the standard deviation).

Altogether, 29,822 high quality *alkB* gene fragment sequences were obtained from 454 sequencing with an average of 828 sequences per sample. As shown in Figure 2, in general evenness of *alkB* genes in all treatments, independent from the sampling time point, was high and ranged from 0.95 to 0.99. Significant differences in evenness were only visible in soil samples where compost C2 has been added, with increased values at the sampling time points 0, 6, and 12 weeks and reduced values after 36 weeks of incubation compared to the other treatments.

**Figure 3:**
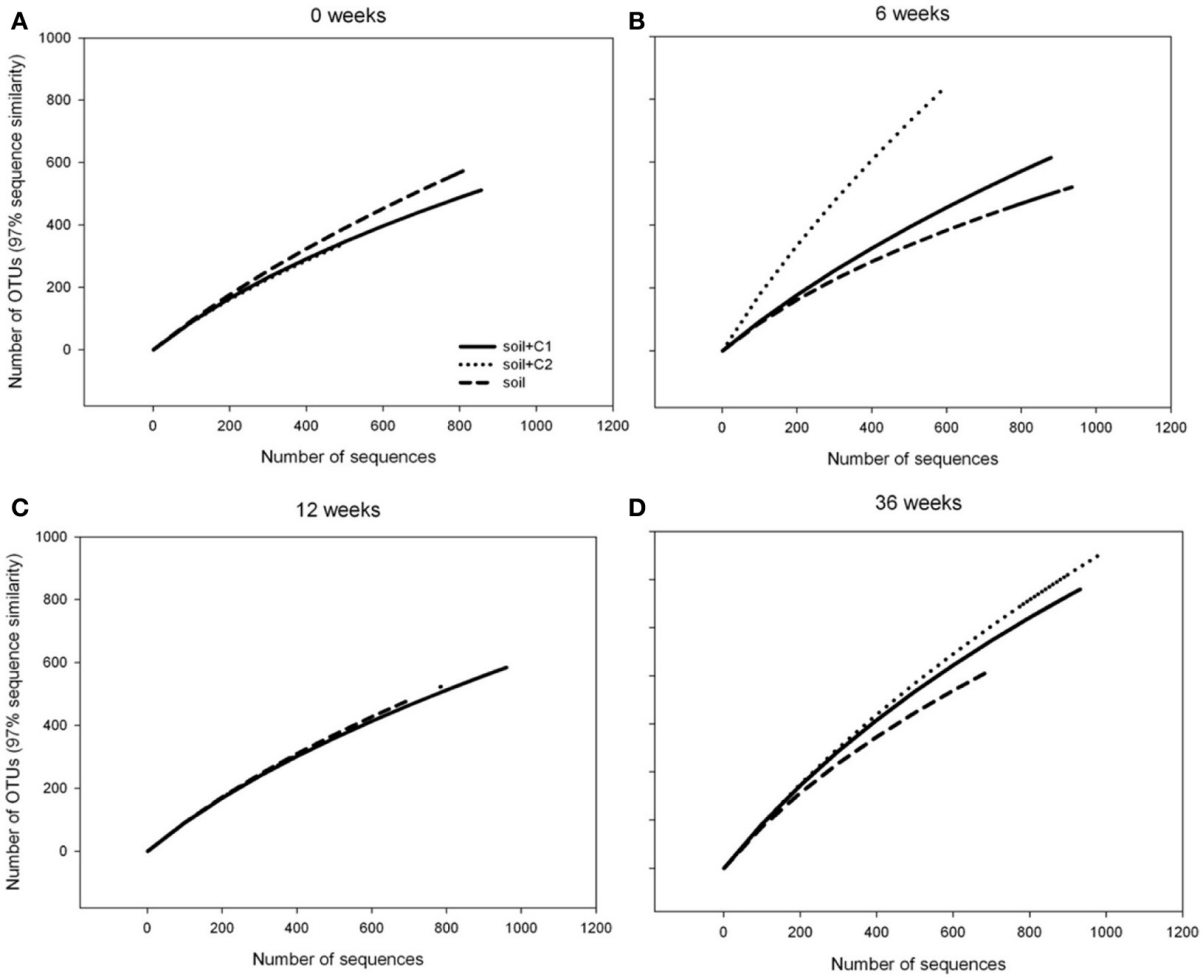
**Rarefaction curves of *alkB* gene fragments for each treatment at different sampling time points**.

Rarefaction curve analysis revealed a constant number of around 400 OTUs in the control samples over time. In contrast an increasing number of OTUs was observed over time in soil samples from treatments where compost C1, respectively, C2 has been added with almost 800 OTUs after 36 weeks of incubation in both treatments. Thus, whereas at the beginning of the experiment the number of sequenced reads was sufficient to reach saturation for all samples, for treatments where compost has been amended after 36 no plateau could be reached when the number of reads was plotted against the number of OTUs (Figure 3).

**Figure 4:**
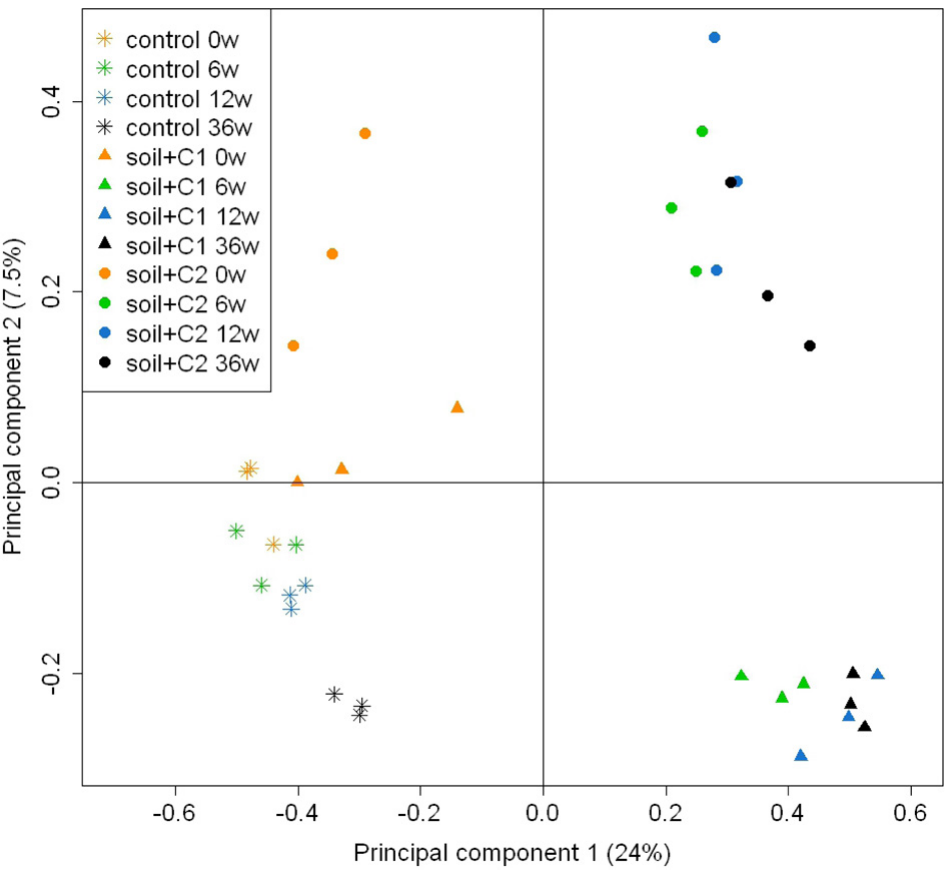
**Principle component analysis based on a dissimilarity matrix of OTUs (>97% sequence identity) data transformed according to Hellinger (Ramette, 2007)**. Only OTUs containing >10 sequences were considered (*n* = 3). The number behind the sample name indicates the incubation time in weeks.

The similarity of *alkB* genes based on a principle component (PC) analysis is visualized in Figure 4. Mainly at later sampling time points PC 1 separates well the control soil from both soils with compost amendments, whereas PC 2 differentiates the soil mixed with old compost (soil + C1) from the soil amended with young compost (soil + C2). PERMANOVA revealed that incubation time as well as the compost type significantly influences the bacterial diversity (both *p*-values < 0.001), Table 1.

**Table 1:** **PERMANOVA results of 454 data analysis**.

	***Df***	**Sum of squares**	**Mean of squares**	***F* Model**	***R***^2^****	***P*-value**
Compost type	2	5.27	2.635	5.43	0.231	0.001
Incubation time	1	2.05	2.050	4.22	0.090	0.001

### Phylogeny of *alkB* harboring soil bacteria

**Figure 5:**
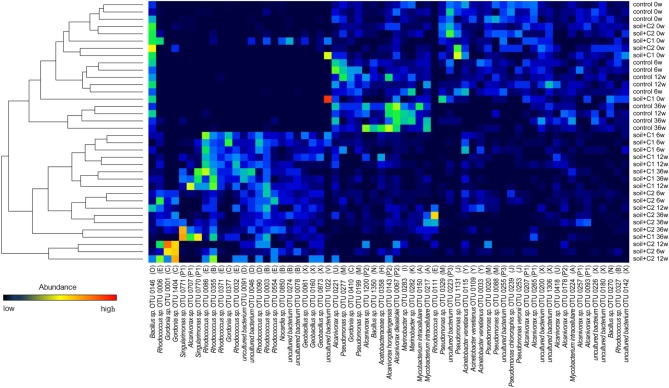
**Heat map of OTUs (containing >50 sequences) that were counted for each sample**. Similarity of the bacterial community for each sample was calculated based on the abundance of each OTU and visualized by a tree. The number after the sample name indicates the incubation time in weeks. The according cluster of each OTU is specified in parentheses.

Out of 29,822 high quality *alkB* gene fragment sequences 9051 OTUs were defined based on 97% sequence identities. For further analysis only those OTUs were considered, that consist of at least two sequences and were longer than 100 amino acids in length. Thus, 3124 representative sequences were included into the phylogenetic analysis using the ARB software. Most of these sequences were assigned to Actinobacteria (30%), followed by y-proteobacteria (19%) and Bacilli (17%). α and β-proteobacteria represented 9 and 1%, respectively, (Table 2). About 24% (749 sequences) of the sequences were assigned to uncultured bacteria. Overall 27 clusters could be defined (Figure 5). The analysis of the generated heatmaps indicated low variability between the replicates. Control soils and soils amended with compost C1 and C2 clustered closely together at day 0. In the control soil only small shifts were visible in the first 6 weeks of incubation. In contrast in samples with compost amendments the structure of *alkB* harboring bacteria changed more pronounced resulting in the formation of clearly separated cluster compared to day 0. Interestingly diversity of *alkB* harboring bacteria was different samples amended with compost C1 or C2.

**Table 2:** **Distribution of *alkB* gene fragments over all samples**.

		**Actinobacteria**		**Bacilli**		**Cytophaga**		**α-proteobacteria**		**y-proteobacteria**		**Unclassified bacteria**		**Total**
Control	0 weeks	33	(1%)	117	(3%)	0		4		84	(3%)	100	(3%)	338
	6 weeks	16	(1%)	87	(3%)	0		17	(1%)	83	(3%)	79	(2%)	282
	12 weeks	15	(1%)	57	(2%)	0		8		71	(2%)	52	(2%)	203
	36 weeks	38	(1%)	28	(1%)	0		21	(1%)	29	(1%)	61	(2%)	177
soil + C1	0 weeks	21	(1%)	53	(2%)	0		6		19	(1%)	50	(2%)	149
	6 weeks	109	(3%)	17	(1%)	0		50	(2%)	63	(2%)	81	(3%)	320
	12 weeks	153	(4%)	5		0		40	(1%)	66	(2%)	34	(1%)	298
	36 weeks	139	(4%)	4		1		37	(1%)	24	(1%)	40	(1%)	245
soil + C2	0 weeks	15	(1%)	117	(3%)	24	(1%)	10		48	(1%)	87	(3%)	301
	6 weeks	123	(4%)	30	(2%)	8		35	(1%)	54	(2%)	65	(2%)	315
	12 weeks	38	(1%)	4		1		5		12		5		65
	36 weeks	232	(8%)	14		3		48	(2%)	39	(1%)	95	(3%)	431
Sum		932	(30%)	533	(17%)	37	(1%)	281	(9%)	592	(19%)	749	(24%)	3124

Only sequences assigned to specific clusters are considered. Numbers in parentheses indicate the percentage of the corresponding class for each sample.

**Figure 6:**
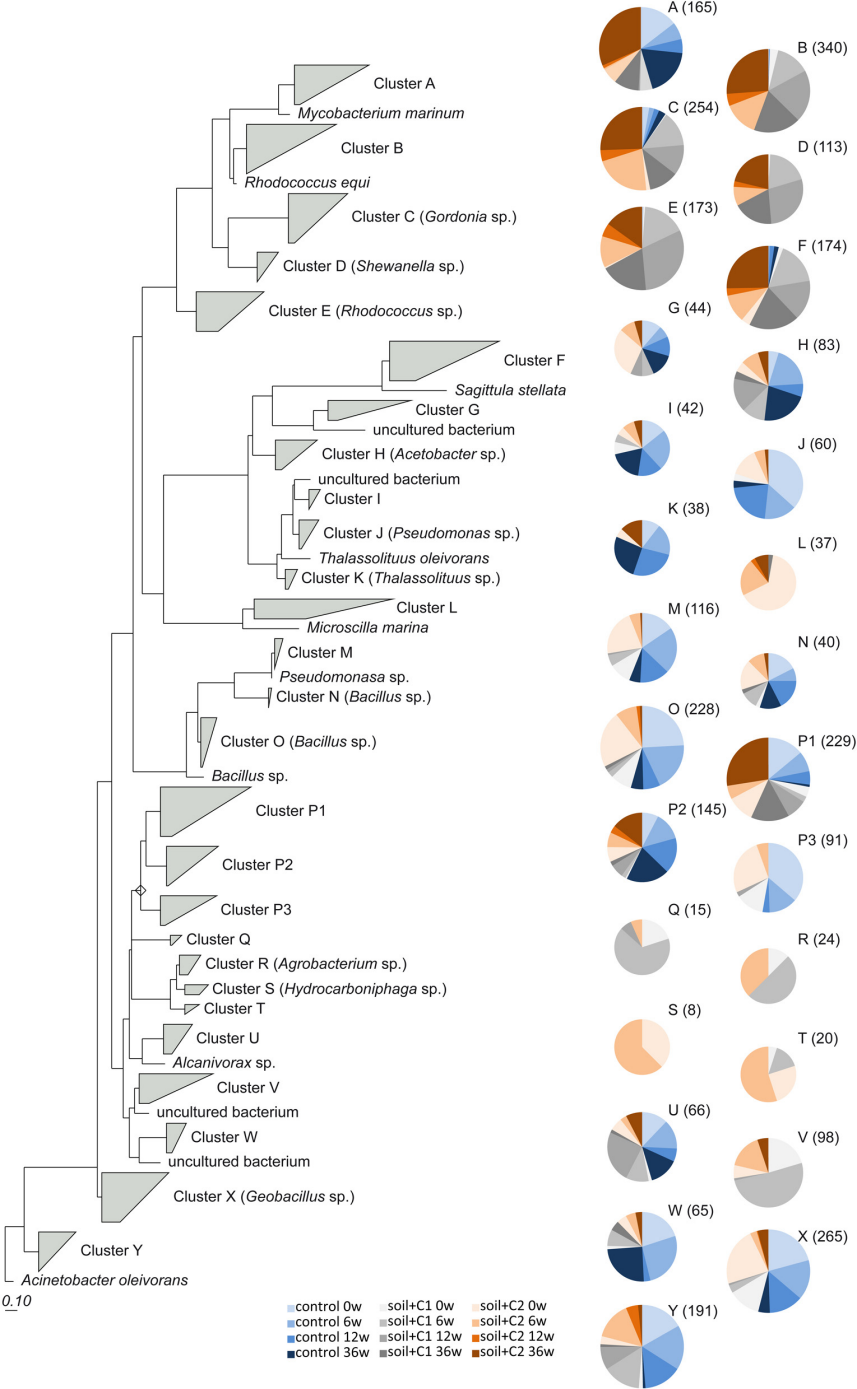
**Phylogenetic tree calculated with the maximum parsimony algorithm based on the reference sequences on amino acids of *alkB* gene fragments provided by RDP**. 454 sequencing amplicons were included. For conciseness, sequences closely related to reference sequences were grouped into clusters from A to Y. Bold numbers indicate the number of sequences within the cluster. If the reference sequence is within the same cluster, the according name is given in parentheses. The number of representative sequences of each sample is indicated with pie charts for each cluster. Different size of pie charts reflects the total number of sequences (given in parentheses) for each cluster.

Sequences detected in the control soil were dominating clusters H (*Acetobacter* sp.), I (uncultured bacteria), J (*Pseudomonas* sp.), K (*Thalassolituus* sp.), M (*Pseudomonas* sp.), N (*Bacillus* sp.), O (*Bacillus* sp.), P2, P3, W (uncultured bacteria), X (*Geobacillus* sp.), and Y (*Acinetobacter* sp.). Overall sequences from the control soil could be found also in six of the other clusters; however here the number of sequences was low (Figure 6) and significantly increased by compost addition [for example sequences assigned to cluster B and E (*Rhodococcus* sp.) or cluster F (*Sagittula* sp.)]. The clusters D (*Shewanella* sp.), L (*Microscilla* sp.), Q, R (*Agrobacterium* sp.), S (*Hydrocarboniphaga* sp.), T and V (uncultured bacteria) contained exclusively sequences detected in soil amended with compost. A specific influence of the different compost types used was only visible for sequences from cluster L and S which were exclusively detected in samples from soil amended with compost C2.

Detailed information about the distribution of the sequences in the phylogenetic tree at the different time points of sampling is summarized in Tables S3, S4. Whereas in control soils at all time point of sampling mainly sequences assigned to Gram positive bacteria related to Bacilli as well as γ-proteobacteria were identified, in soil samples amended with compost C1 and C2 the maximal number of representative sequences could be assigned to Actinobacteria after 6 weeks of incubation at all later sampling time points. In soil samples amended with compost C2 also a remarkable high number of sequences associated to Cytophaga mainly at the time point after compost addition could be identified, which was significantly reduced at the other sampling time points.

## Discussion

In this study we analyzed in a microcosm experiment the effects of the amendment of different composts with contrasting maturation stage on the abundance and diversity of *alkB* harboring bacteria in a soil contaminated with hydrocarbons. Previous studies revealed that *alkB* related sequences are present in a wide range of soil and water derived bacteria, such as *Acinetobacter*, *Alcanivorax*, *Burkholderia*, *Mycobacterium*, *Pseudomonas*, *Rhodococcus* and others (Van Beilen and Funhoff, 2007; Pérez-De-Mora et al., 2011). Furthermore, many *alkB* harboring bacteria (e.g., *Mycobacterium and Nocardia*) carry in addition to the *alk* operon genes like *almA* which encodes for an enzyme that is involved in degrading of alkanes with a chain length of C_32_ and longer (Feng et al., 2007). Thus, *alkB* seems to be not only a good marker to study the potential to degrade alkanes with a chain length between C5 and C16 (Van Beilen and Funhoff, 2005; Rojo, 2009), but also the general indicator for alkane degradation in the environment.

However, the number of *alkB* harboring operons per cell differs between different bacterial species. For bacteria of the genus *Rhodococcus* up to seven operons per cell have been described, whereas for other bacteria (e.g., Pseudomonads) the number of operons/cell was between 1 and 2 (Heiss-Blanquet et al., 2005). This must be taken into account when the results of this study are interpreted mainly in terms of increasing abundance of *alkB* harboring bacteria in the response of compost addition.

Obviously compost may have two different effects. On the one hand it might provide a suitable substrate to stimulate the growth of microbes from the original soil capable to degrade alkanes, like it was shown for Actinobacteria, which start to become highly abundant 6 weeks after compost amendment independent from the type of compost. As in addition no shift in diversity of this group was observed, we may assume here a growth stimulation effect. Here our results correspond to other observations, that litter material stimulates the abundance of *alkB* harboring communities in soil as well induces changes in diversity (Schulz et al., 2012). Furthermore, the introduced amounts of nutrients may stimulate co-degradation of alkanes in soil and changes sorption properties of alkanes to soil particles. For example several *Pseudomonas* species in soil are well known for producing biosurfactants like rhamnolipids (*P. aeruginosa* Kumar et al., 2008, *P. fluorescens* Abouseoud et al., 2008) that enhance hydrocarbon uptake through emulsification via decreasing the surface tension and forming micelles (Das and Chandran, 2011).

On the other hand, for other groups mainly Cytophaga related bacteria an effect was only visible directly after compost addition, indicating that the introduced microbes by compost were not able to survive in the soil environment. In contrast other introduced bacteria, mainly from related to proteobacteria could obviously successfully establish in soil. Sequencing of *alkB* gene fragments revealed that those organisms are closely related to common soil bacteria like members of *Agrobacterium*. Some of the representative sequences are closely related to genera known for their appearance in alkane rich environments. For example a number of sequences obtained from soil samples amended with compost clustered around the genus *Hydrocarboniphaga*, a member of γ-proteobacteria. (Palleroni et al., 2004) cultivated the strain *Hydrocarboniphaga effusa* DSM 16095^T^ successfully that originally was isolated from soil contaminated with heavy fuel oil hydrocarbons in New Jersey. Recently, Liu et al. (2011) linked another strain, *Hydrocarboniphaga daqingensis* NBRC 104238^T^ to degradation of alkanes with medium chain length (C_9_–C_17_). Additionally, some representative sequences from soils with amendment of C1 or C2 could be linked to the genus *Thalassolituus*, which is closely related to *alkB* sequences of Pseudomonas. *T. oleivorans* strain DSM 14913, a strain able degrade alkanes in marine environments described by Yakimov et al. (2004) and recently fully sequenced (Golyshin et al., 2013).

Also for most other *alkB* sequences found in our study which were partly being present also in the original soil samples without compost amendment like *Singularimonas variicoloris*, *Mycobacterium* sp. *Nocardia* sp. *Pseudomonas veronii* or *Acinetobacter venetianus* alkane degradation has been proven (Onaca et al., 2007; Friedrich and Lipski, 2008; Luckarift et al., 2011; Wang and Shao, 2013). Some of them (e.g., *Mycobacterium and Nocardia*) harbor in addition to the *alk* operon genes like *almA* which encodes for an enzyme that is involved in degrading of alkanes with a chain length of C_32_ and longer (Feng et al., 2007).

Although our study clearly indicates that the amendment of composts to petroleum contaminated soils have pronounced effects of the abundance and diversity of *alkB* harboring bacteria, future studies must prove that this concept will also lead an enhanced stimulation of alkane degraders (based on mRNA analysis) and subsequently to enhanced biodegradation rates of alkanes in the particular soils. In addition it is well known that mainly not well maturated composts contain a large number of bacteria with (human) pathogenic potential. As our study could prove that probably some bacteria from compost are able to colonize the soil matrix, thus more data is needed also on this aspect before the method of compost application for bioremediation can be applied on a broader range.

### Conflict of interest statement

The authors declare that the research was conducted in the absence of any commercial or financial relationships that could be construed as a potential conflict of interest.

## Supplementary material

Table S1 **Summary of sequence processing**.

Table S2 **Average total alkane concentrations (mg/kg) with standard errors (*n* = 3)**. Different letters indicate statistically significant difference between averages according to Tukey's multiple comparison test (*P* ≤ 0.05).

Table S3 ***alkB* gene fragments of amplicon sequencing were included into a phylogenetic ARB tree on amino acid level**. Sequences closely related to reference sequences were grouped to clusters. Dashes (–) indicate sequences that could not be assigned to any specific cluster.

Table S4 **Distribution of representative sequences within each cluster for each sample as shown in the pie charts presented in Figure 6**. Numbers are given in percentages.
