# Supplementary material for: Effects of different compost amendments on the abundance and composition of alkB harboring bacterial communities in a soil under industrial use contaminated with hydrocarbons
Source: Front Microbiol. 2014 Mar 13;5:96. doi: 10.3389/fmicb.2014.00096 (PMC3952045; doi:10.3389/fmicb.2014.00096)
Supplement: Table S1 — Summary of sequence processing. [file DataSheet1.PDF]

Supplemental information

Table S1: Summary of sequence processing.

average initial sequence read length (bp)	493.8
total number of reads	402,154
number of reads after trimming	89,244
unique sequence reads after trimming	58,473

Table S2: Average total alkane concentrations (mg /kg) with standard errors (n=3). Different letters indicate statistically significant difference between averages according to Tukey´s multiple comparison test ($P \leq 0.05$).

Treatment	Time	Conc. mg/kg		
Soil	0 weeks	854	±19	a
	12 weeks	642	±32	b
Soil + C1	0 weeks	854	±17	a
	12 weeks	537	±24	c
Soil + C2	0 weeks	795	±15	a
	12 weeks	502	±23	c

Table S3: *alkB* gene fragments of amplicon sequencing were included into a phylogenetic ARB tree on amino acid level. Sequences closely related to reference sequences were grouped to clusters. Dashes (–) indicate sequences that could not be assigned to any specific cluster

Taxon identified	Cluster	Number of representative sequences
Acetobacteraceae sp.	H	83
Acetobacteraceae sp.	-	11
Acidisphaera sp.	-	1
Acinetobacter sp.	Y	191
Aeromicrobium sp.	-	23
Agrobacterium sp.	R	24
Agrobacterium sp.	-	24
Alcanivorax sp.	U	66
Alcanivorax sp.	-	9
Bacillus sp.	N	40
Bacillus sp.	O	228
Bradyrhizobiacae sp.	-	1
Caulobacter sp.	-	5
Cluster P1	P1	229
Cluster P2	P2	145
Cluster P3	P3	91
Cluster Q	Q	15
Cluster T	T	20
Conexibacter sp.	-	4
Dietzia sp.	-	1
Geobacillus sp.	X	265
Gordonia sp.	C	254
Gordonia sp.	-	29
Hydrocarboniphaga sp.	S	8

Methylibium sp.	-	6
Microscilla sp.	L	37
Mycobacterium sp.	A	165
Mycobacterium sp.	-	58
Nocardia sp.	-	27
Nocardioides sp.	-	4
Pedobacter sp.	-	4
Pseudomonas sp.	J	60
Pseudomonas sp.	M	116
Pseudomonas sp.	-	26
Pseudoxanthomonas sp.	-	4
Ralstonia sp.	-	27
Rhodococcus sp.	B	340
Rhodococcus sp.	E	173
Rhodococcus sp.	-	100
Rhodopseudomonas sp.	-	2
Sagittula sp.	F	174
Sagittula sp.	-	3
Shewanella sp.	D	113
Stenotrophomonas sp.	-	16
Thalassolituus sp.	K	38
uncultured bacterium	G	44
uncultured bacterium	I	42
uncultured bacterium	V	98
uncultured bacterium	W	65
uncultured bacterium	-	100
Xanthobacter sp.	-	6

Table S4: Distribution of representative sequences within each cluster for each sample as shown in the pie charts presented in Figure 6. Numbers are given in percentages.

			**soil**				**soil+C1**				**soil+C2**				
**Taxon identified**	**Cluster**	**Number of representative sequences**	**0w**	**6w**	**12w**	**36w**	**0w**	**6w**	**12w**	**36w**	**0w**	**6w**	**12w**	**36w**	**sum**
Acetobacter sp.	H	83	4.8	19.3	6.0	21.7	0.0	10.8	15.7	3.6	4.8	8.4	0.0	4.8	100
Acinetobacter sp.	Y	191	16.8	17.3	14.7	1,0	1.6	14.7	8.9	1.0	3.1	14.7	4.7	1.6	100
Agrobacterium sp.	R	24	0.0	0.0	0.0	0,0	12.5	50.0	0.0	0.0	0.0	37.5	0.0	0.0	100
Alcanivorax sp.	U	66	12.1	13.6	6.1	13,6	1.5	10.6	24.2	1.5	6.1	3.0	0.0	7.6	100
Bacillus sp.	N	40	17.5	7.5	17.5	12,5	2.5	10.0	0.0	2.5	17.5	10.0	0.0	2.5	100
Bacillus sp.	O	228	24.1	18.9	6.6	4,8	8.3	2.2	1.3	1.3	21.9	8.3	1.3	0.9	100
Cluster P1	P1	229	14.0	7.9	5.2	0,9	3.9	2.2	7.9	14.8	10.5	5.2	0.0	27.5	100
Cluster P2	P2	145	7.6	13.1	16.6	20,0	0.7	2.1	6.2	2.1	6.9	6.9	3.4	14.5	100
Cluster P3	P3	91	36.3	13.2	3.3	0,0	13.2	0.0	2.2	0.0	26.4	5.5	0.0	0.0	100
Cluster Q	Q	15	0.0	0.0	0.0	0,0	20.0	66.7	6.7	0.0	0.0	6.7	0.0	0.0	100
Cluster T	T	20	0.0	0.0	0.0	0,0	5.0	15.0	0.0	0.0	25.0	55.0	0.0	0.0	100
Geobacillus sp.	X	265	20.8	15.5	13.2	4,5	12.5	3.0	0.8	0.0	22.6	2.6	0.4	4.2	100
Gordonia sp.	C	254	2.8	2.0	2.0	2,8	0.4	13.8	11.8	11.4	1.6	21.7	4.3	25.6	100
Hydrocarboniphaga sp.	S	8	0.0	0.0	0.0	0,0	0.0	0.0	0.0	0.0	37.5	62.5	0.0	0.0	100
Microscilla sp.	L	37	0.0	0.0	0.0	0,0	0.0	0.0	0.0	2.7	64.9	21.6	2.7	8.1	100
Mycobacterium sp.	A	165	14.5	6.7	5.5	18,8	4.8	0.0	0.6	9.7	6.1	0.6	1.2	31.5	100
Pseudomonas sp.	J	60	36.7	15.0	21.7	3,3	3.3	0.0	0.0	0.0	13.3	5.0	0.0	1.7	100
Pseudomonas sp.	M	116	15.5	21.6	13.8	5,2	10.3	5.2	0.9	0.0	21.6	5.2	0.0	0.9	100

Rhodococcus sp.	B	340	0.3	0.0	0.3	0.0	3.2	13.2	20.3	18.2	0.0	13.5	4.7	26.2	100
Rhodococcus sp.	E	173	0.6	0.0	0.0	0.0	0.6	16.8	30.6	18.5	0.6	12.1	5.2	15.0	100
Sagittula sp.	F	174	0.0	0.6	1.7	1.7	1.7	16.7	15.5	19.5	3.4	10.9	2.9	25.3	100
Shewanella sp.	D	113	0.0	0.0	0.0	0.0	0.9	19.5	28.3	18.6	0.0	8.8	2.7	21.2	100
Thalassolituus sp.	K	38	10.5	18.4	26.3	26.3	0.0	0.0	0.0	0.0	5.3	0.0	0.0	13.2	100
uncultured bacterium	G	44	11.4	6.8	11.4	13.6	0.0	6.8	6.8	0.0	29.5	9.1	0.0	4.5	100
uncultured bacterium	I	42	14.3	23.8	14.3	19.0	7.1	4.8	0.0	0.0	4.8	7.1	0.0	4.8	100
uncultured bacterium	V	98	0.0	0.0	0.0	0.0	20.4	51.0	1.0	0.0	6.1	16.3	0.0	5.1	100
uncultured bacterium	W	65	20.0	26.2	3.1	24.6	1.5	7.7	0.0	4.6	4.6	4.6	0.0	3.1	100
